# Complete genome sequence of a methanotrophic bacterium, *Methylomicrobium lacus* strain 22M6SE5-12 isolated from a freshwater sediment environment

**DOI:** 10.1128/mra.01006-24

**Published:** 2024-11-29

**Authors:** Eui-Jin Kim, Kyung June Yim, Mi-Jung Bae, Kook-IL Han

**Affiliations:** 1 Freshwater Biodiversity Research Bureau, Nakdonggang National Institute of Biological Resources (NNIBR), Sangju, South Korea; DOE Joint Genome Institute, Berkeley, California, USA

**Keywords:** complete genome, *Methylomicrobium lacus*, methanotorph, freshwater sediment

## Abstract

We assembled the complete genome of *Methylomicrobium lacus* strain 22M6SE5-12 isolated from a freshwater sediment in South Korea. The genome consists of a 4.36-Mbp chromosome and two plasmids and has 3,961 coding sequences, 12 rRNA genes, and 55 tRNA genes. We identified a gene set encoding the particulate methane monooxygenase subunits.

## ANNOUNCEMENT

Methanotrophs are specialized bacteria that use methane (CH₄) as their primary carbon and energy source (1). These microorganisms play an important role in global carbon cycling by consuming CH₄, a potent greenhouse gas, and converting it into biomass and CO₂ (2). They are found in diverse habitats, including freshwater and marine environments, soils, wetlands, and even extreme habitats, such as hot springs (3), and have gained increasing research attention because of their potential applications (4).

**Fig 1:**
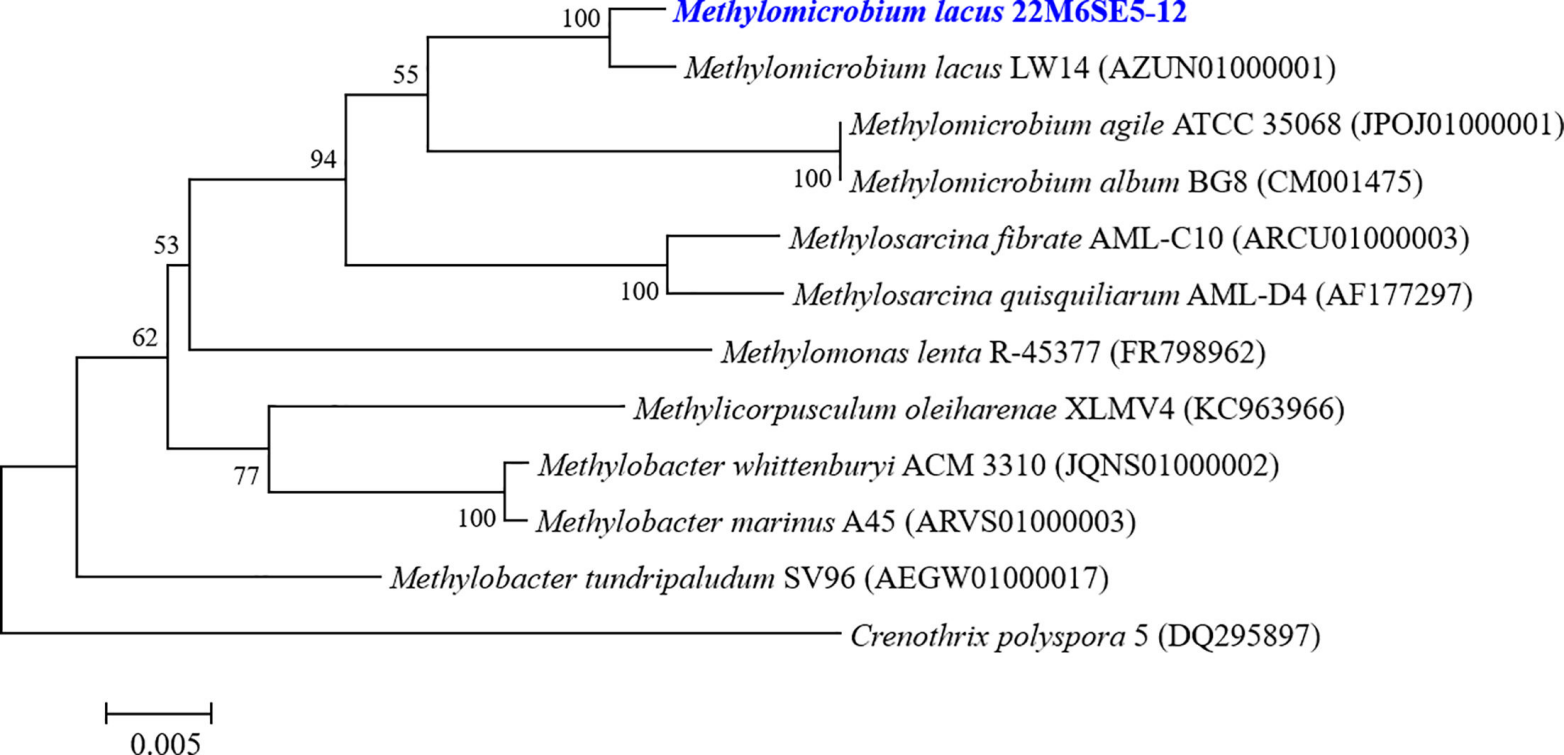
Phylogenetic tree of *M. lacus* 22M6SE5-12 based on the sequences for 16S rRNA gene marker. The tree is depicted to scale, with branch lengths represented in the same units as the evolutionary distances used to construct the phylogenetic tree. For the alignment of genetic sequences, the EZ editor ver. 2 (14) program was used. The tree was built and analyzed using MEGA11 software (15). The neighbor-joining method was applied for statistical analysis, with 1,000 bootstrap replicates to ensure robustness.

*Methylomicrobium lacus* is a Gram-negative, non-motile, gamma-proteobacterial methanotroph capable of growing on CH₄ (5). This species possesses particulate methane monooxygenase (pMMO) and lacks soluble MMO (6). We describe the complete genome sequence and annotation of *M. lacus* strain 22M6SE5-12 (Fig. 1), which was isolated from a freshwater sediment in Sangju-si, Korea (36°25'8.47"N; 128°14'9.59"E). The sediment sample (1 g), collected on August 19, 2022, was serially diluted in saline (0.85% NaCl) and incubated at 25°C in 250 mL vials containing 50 mL of nitrate mineral salt (NMS) minimal medium (7) under shaking at 150 rpm in a CH₄/air atmosphere (3:7) for 2 weeks. The cultures were transferred to a solid NMS medium containing 1.5% (wt/vol) Difco agar and incubated under the same CH₄/air atmosphere for 3–7 days to isolate a single colony. The genomic DNA was purified from the strain grown on the NMS medium by using the Maxwell RSC Tissue DNA kit (Promega). Whole-genome sequencing was performed on sample 22M6SE5-12, using the PacBio Sequel and Illumina NovaSeq 6000. For PacBio sequencing, gDNA was sheared with a g-TUBE device (Covaris), and a library (N50 = 9,514, average length = 7,228, total subreads = 101,534) was constructed with a size selection range of 7–12 kb by using the 15 kb SMRTbell template preparation kit (Pacific Biosciences). Alternatively, a short read library (5,624,144 reads, Q30(%) =98.45) was prepared using the TruSeq Nano DNA kit (Illumina). The sequencing data were subjected to *de novo* assembly, error correction, and annotation. All tools were applied with default parameters unless noted otherwise. The genome was assembled using Canu (v1.7) for PacBio data (8). Post-assembly correction was performed by aligning the Illumina reads to the PacBio-assembled genome using Pilon (v1.21) (9). The genome size was estimated using K-mer analysis, and heterozygosity and repeat content were assessed using Jellyfish (v2.2.10) and GenomeScope (10, 11). For assembly quality assessment, we used BUSCO (v5.1.3) (12). Coding sequences (CDSs), transfer RNA (tRNA), and ribosomal RNA (rRNA) genes were annotated by the NCBI Prokaryotic Genome Annotation Pipeline (PGAP) (13).

The genome comprised 3,961 CDSs, 55 tRNA genes, and 12 rRNA genes across the assembled contigs (Table 1). Although the type strain *M. lacus* LW14^T^ has a single chromosome (6), *M. lacus* 22M6SE5-12 has a 4.36-Mbp chromosome and two plasmids (67 and 58 kbp, respectively) (Table 1). As a typical obligate methanotroph, *M. lacus* 22M6SE5-12 produces pMMO for CH₄ oxidation. We identified a gene set encoding the pMMO subunits (PmoA, PmoB, and PmoC) in the chromosome sequence, but we found no evidence of soluble MMO genes.

**TABLE 1:** Assembly and annotation statistics for *M. lacus* 22M6SE5-12

Parameter	Chromosome	Plasmid 1	Plasmid 2	Total
Length (bp)	4,366,859	67,659	58,488	4,493,006
GC content (%)	55.4	49.3	51.2	55.3
CDSs (N)	3,827	65	69	3,961
rRNAs (N)	12	0	0	12
tRNAs (N)	55	0	0	55
Sequencing depth (×)	137.8	371.7	356.3	144.1
GenBank accession no.	CP169217	CP169218	CP169219	

## Data Availability

The whole genome sequence of strain 22M6SE5-12 has been deposited in GenBank under accession number CP169217, CP169218, and CP169219, in BioProject under accession number PRJNA1153416, and in BioSample under accession number SAMN43359730. The corresponding SRA accession numbers are SRX26228384 (PacBio) and SRR30827936 (Illumina), respectively.
